# Apoptosis of osteosarcoma cultures by the combination of the cyclin-dependent kinase inhibitor SCH727965 and a heat shock protein 90 inhibitor

**DOI:** 10.1038/cddis.2013.101

**Published:** 2013-03-28

**Authors:** W Fu, S S Sharma, L Ma, B Chu, M M Bui, D Reed, W J Pledger

**Affiliations:** 1 Department of Molecular Oncology, Gonzmart Research Laboratory, and the Sarcoma Research Program, H. Lee Moffitt Cancer Center and Research Institute, Tampa, FL, USA; 2 Department of Experimental Therapeutics, the Sarcoma Research Program, H. Lee Moffitt Cancer Center and Research Institute, Tampa, FL, USA; 3 Department of Sarcoma, the Sarcoma Research Program, H. Lee Moffitt Cancer Center and Research Institute, Tampa, FL, USA

**Keywords:** osteosarcoma, cyclin-dependent kinase, heat shock protein 90, apoptosis, SCH727965, NVP-AUY922

## Abstract

Osteosarcoma (OS) is an aggressive bone cancer typically observed in adolescents and young adults. Metastatic relapse accounts primarily for treatment failure, and obstacles to improving cure rates include a lack of efficacious agents. Our studies show apoptosis of OS cells prepared from localized and metastatic tumors by a novel drug combination: SCH727965 (SCH), a cyclin-dependent kinase inhibitor, and NVP-AUY922 (AUY) or other heat shock protein 90 inhibitor. SCH and AUY induced apoptosis when added simultaneously to cells and when AUY was added to and removed from cells before SCH addition. Sequential treatment was most effective when cells received AUY for ∼12 h and when SCH was presented to cells immediately after AUY removal. The apoptotic protein Bax accumulated in mitochondria of cotreated cells but was primarily cytosolic in cells receiving either agent alone. Additional data show that SCH and AUY cooperatively induce the apoptosis of other sarcoma cell types but not of normal osteoblasts or fibroblasts, and that SCH and AUY individually inhibit cell cycle progression throughout the cell cycle. We suggest that the combination of SCH and AUY may be an effective new strategy for treatment of OS.

Osteosarcoma (OS) is the most common primary bone malignancy. It affects people of all ages, with peak incidence in adolescents and young adults.^1^ It occurs most commonly in the long bones of the limbs and metastasizes primarily to the lungs. The 5-year overall survival rate for patients with localized disease is ∼60%, for patients with metastatic spread, it is less than 30% these rates have not improved significantly in the past 30 years.^2^ Current treatment includes complete surgical excision, which is required for a cure, and chemotherapy, most notably a combination of doxorubicin, methotrexate, and cisplatin (collectively referred to as MAP) and sometimes ifosfamide.^1, 3^ Chemotherapy typically causes tumor necrosis; although amount of necrosis correlates with outcome, even patients with excellent necrosis face a relapse risk of 20%. Thus, there is a need for new therapies for patients with metastatic and relapsed OS.

Towards this goal, we previously showed apoptosis of established OS cell lines (e.g., U2OS and SaOs-2) by the cyclin-dependent kinase (CDK) inhibitor SCH727965 (SCH) (also known as dinaciclib).^4^ SCH inhibits the activity of CDK1, CDK2, CDK9 and CDK5.^5, 6^ CDK1 and CDK2 promote cell cycle progression, whereas CDK9 facilitates transcriptional elongation; CDK5 functions primarily in neurons. Our study also showed sensitivity, albeit modest, of newly prepared OS cell cultures to SCH. It is possible that these cultures will respond more robustly to SCH in combination with other inhibitors.

Heat shock protein (Hsp) 90 is an ATP-driven molecular chaperone.^7, 8^ It helps nascent polypeptides assume biologically active conformations, disrupts protein aggregates and repairs or removes misfolded proteins. In cancer cells, it drives oncogenesis by stabilizing labile oncoproteins and by maintaining homeostasis in hostile environments. More than 200 proteins (termed ‘clients') interact with Hsp90; these include nuclear steroid receptors, transcription factors and kinases (e.g., AKT), many of which occupy central positions in biological networks.^9^ Hsp90 is a dimer that requires ADP/ATP exchange and interaction with cochaperones for activity.^10^

Hsp90 inhibitors include 17-allylamino-17-demethoxygeldanamycin (AAG),^11^ 17-(2-dimethylaminoethyl) amino-17-demethoxygeldanamycin (DMAG),^12^ NVP-HSP990 (HSP990)^13^ and NVP-AUY922 (AUY).^14^ All interact with the ATP binding site in the N terminus of Hsp90 and short circuit the Hsp90 chaperone cycle; this results in the proteasome-mediated degradation of Hsp90 clients and the consequent collapse of multiple signaling pathways.^10^ Hsp90 inhibitors have a 100-fold greater affinity for Hsp90 in tumor cells than in normal cells and thus are selective for tumor cells.^15^ AAG and DMAG were the first Hsp90 inhibitors tested in clinical trials; however, both are problematic due to toxicity, and AAG has poor aqueous solubility.^16, 17^ The synthetic inhibitors HSP990 and AUY are currently in clinical trials.

In this study, we examined the effects of SCH and Hsp90 inhibitors on the survival of OS cell cultures prepared by us from resected OS tumors. Drugs were added to OS cells at the same time or at separate times, and their effects on the viability of normal osteoblasts was also determined. Our results suggest that the combination of SCH and Hsp90 inhibitor may be useful for treating OS.

## Results

### Apoptosis of OS cells by the combination of SCH and Hsp90 inhibitor

OS cell cultures were prepared from four resected tumors (designated OS1001, OS1002, OS1003, OS1004) as described in Materials and Methods. OS1001 was a localized tumor in a young adult. It showed excellent necrosis (>90%) after MAP chemotherapy, and the patient remains disease-free more than 2 years from diagnosis. OS1003 and OS1004 were primary tumors in young adult patients with widespread and non-resectable metastatic disease at presentation. OS1003 and OS1004 received MAP chemotherapy in the neoadjuvant setting with 41% and 90% necrosis, respectively; a biopsy specimen of a metastasis of OS1003 had 88% necrosis. The patients lived for 7 (OS1003) and 10 (OS1004) years from diagnosis after progressing though adjuvant MAP and additional chemotherapies. OS1002 was a previously treated, recurrent metastatic tumor without systemic therapy after recurrence; the patient has been lost to follow-up.

**Figure 1:**
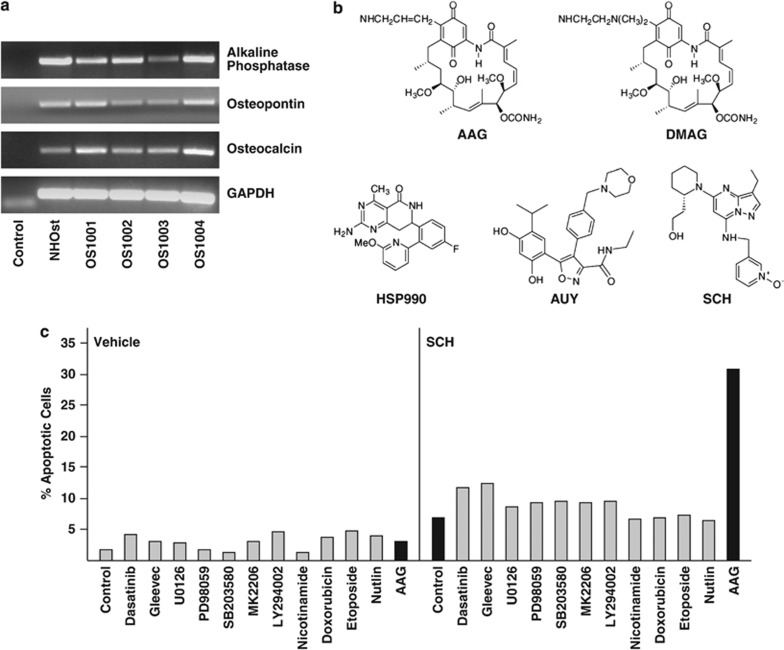
Cell lines and inhibitors. (**a**) RT-PCR was performed on cDNA derived from the indicated cell lines using probes for alkaline phosphatase, osteopontin, osteocalcin and GAPDH (loading control). NHOst cells are normal human osteoblasts and are included as a positive control. Control (lane 1) has no cDNA. (**b**) Structures of the inhibitors used in this study. (**c**) OS1002 cells received 10 nℳ SCH and the indicated inhibitors for 72 h. Percent apoptotic cells was determined by FACS analysis of annexin V-stained cells. Concentrations of inhibitors used were: MK2206 (1 *μ*ℳ); Gleevec (10 *μ*ℳ); etoposide (1 *μ*ℳ); doxorubicin (250 nℳ); dasatinib (100 nℳ); LY294002 (20 *μ*ℳ); SB203580 (10 *μ*ℳ); U0126 (10 *μ*ℳ); PD98059 (50 *μ*ℳ), AAG (1 *μ*ℳ); nicotinamide (100 nℳ); and nutlin (5 *μ*ℳ). Black bars denote cells receiving AAG, SCH or both

All four OS cultures expressed osteoblastic markers (osteopontin, osteocalcin and alkaline phosphatase) as determined by RT-PCR (Figure 1a). Normal human osteoblasts (NHOst) are shown as a positive control. The structures of SCH and of the Hsp90 inhibitors used in our study are diagramed in Figure 1b.

We previously showed that newly prepared OS cultures apoptose weakly when exposed to SCH alone for 72–96 h.^4^ To potentially improve this response, we cotreated OS1002 cells with 10 nℳ SCH and inhibitors of tyrosine kinases (dasatinib, Gleevec), mitogen-activated protein kinases (U0126, PD98059, SB203580), AKT signaling (MK2206, LY294002), class III histone deacetylases (nicotinamide), DNA synthesis (doxorubicin, etoposide), p53 (nutlin) or Hsp90 (AAG) for 72 h. Apoptosis was monitored by FACS (fluorescence-activated cell sorting) analysis of annexin V-stained cells; annexin V detects externalized phosphatidylserines, a marker of early apoptosis.^18^ None of the agents tested induced apoptosis in the absence of SCH, and only AAG increased responsiveness to SCH (Figure 1c). AAG by itself had little effect if any. Thus, Hsp90 inhibitors represent a means of sensitizing OS cells to SCH (or *vice versa*).

**Figure 2:**
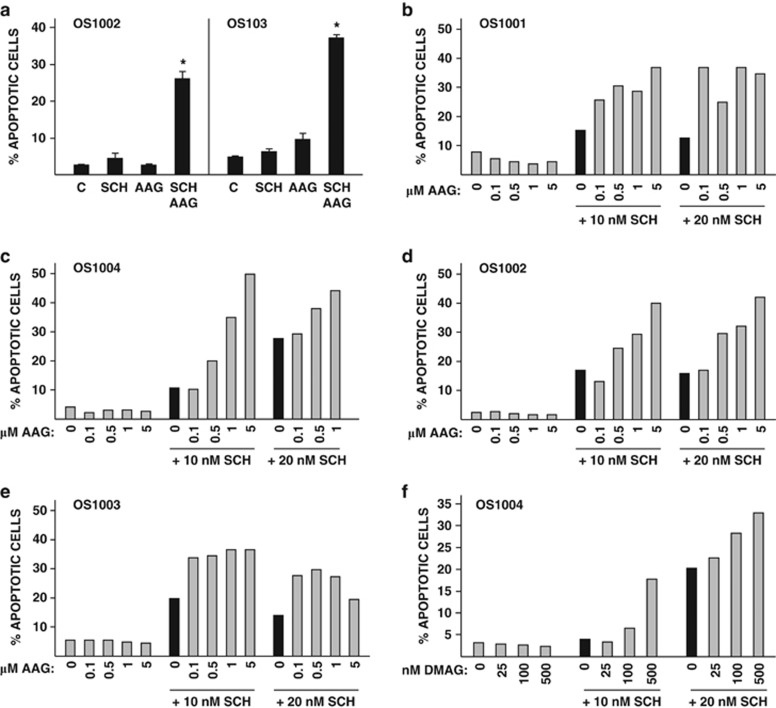
Apoptosis of OS cells cotreated with SCH and AAG. (**a**) OS1002 and OS1003 cells received 10 nℳ SCH, 5 mℳ AAG, or both for 48 h. Error bars indicate S.D. Asterisk indicates significant *P*-value. *P*-values for OS1002 cells are: SCH, 0.15; AAG, 0.59; both, 0.0019. *P*-values for OS1003 cells are: SCH, 0.14; AAG, 0.05; both, 0.0003. C: control. (**b–e**) Cells received the indicated concentrations of SCH and AAG for 48 h. Black bars denote cells receiving SCH alone. (**f**) OS1004 cells received the indicated concentrations of SCH and DMAG for 48 h. Black bars denote cells receiving SCH alone. (**a–f**) Percent apoptotic cells was determined by FACS analysis of annexin V-stained cells

The apoptotic response of OS cells to SCH plus AAG was highly signficant (Figure 2a) and was seen in all four OS cell lines (Figure 2b). Percentages of cells undergoing apoptosis were generally less than 10% for AAG, less than 20% for SCH, and more than 30% and as high as 50% for SCH plus AAG. Concentrations of AAG eliciting maximal responses in combination with SCH ranged from 0.1 *μ*ℳ to 5 *μ*ℳ (Figures 2b–e). DMAG (500 nℳ) also induced apoptosis when added to cells with SCH (Figure 1f). Some cotreated cells were both annexin V-positive and propidium iodide-positive, which is indicative of late apoptosis (‘secondary necrosis') or of necrotic, non-apoptotic cell death (Supplementary Figures 1a–d).^19^

**Figure 3:**
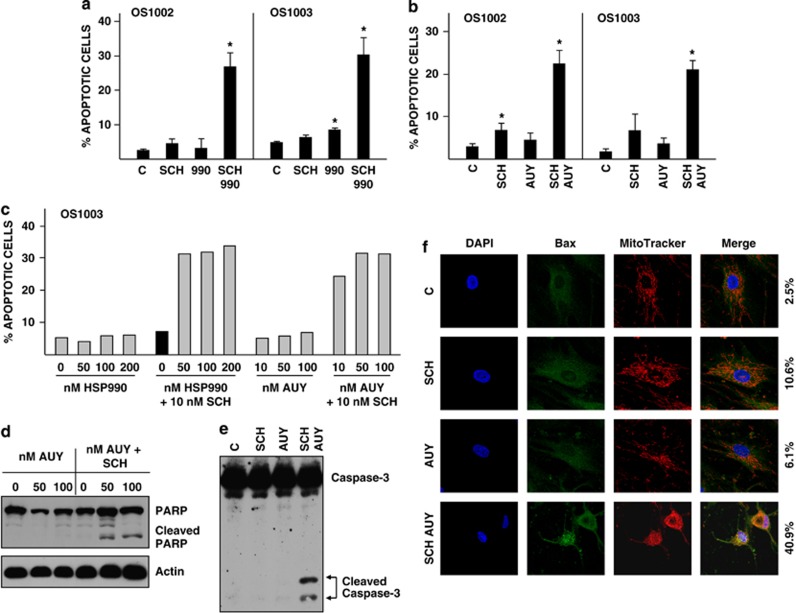
Caspase-3 activation and Bax translocation in OS cells cotreated with SCH and AUY. (**a**) OS1002 and OS1003 cells received 10 nℳ SCH, 100 HSP990 (990), or both for 48 h. Error bars indicate S.D. Asterisk indicates significant *P*-value. *P*-values for OS1002 cells are: SCH, 0.15; 990, 0.23; both, 0.007. *P*-values for OS1003 cells are: SCH, 0.14; 990, 0.01; both, 0.01. C: control. (**b**) OS1002 and OS1003 cells received 10 nℳ SCH and 100 nℳ AUY or both for 48 h. Error bars indicate S.D. Asterisk indicates significant *P*-value. *P*-values for OS1002 cells are: SCH, 0.008; AUY, 0.05; both, 0.001. *P*-values for OS1003 cells are: SCH, 0.22; AUY, 0.09; both, 0.0002. C: control. (**c**) OS cells received the indicated concentrations of inhibitors for 48 h. (**a–c**) Percent apoptotic cells was determined by FACS analysis of annexin V-stained cells. Black bars denote cells receiving SCH (**d**) OS1002 cells received 10 nℳ SCH and 50 or 100 nℳ AUY for 48 h. Cell extracts were western blotted with antibody to PARP or actin (loading control). (**e**) OS1002 cells received 10 nℳ SCH and 100 nℳ AUY for 24 h. Cell extracts were western blotted with antibody to caspase-3. C: control. (**f**) OS1003 cells received 10 nℳ SCH and 100 nℳ AUY for 48 h. Cells were co-stained with anti-Bax antibody (green), MitoTracker Red (red), and the nuclear marker DAPI (blue) as described in Materials and Methods. Magnification is × 1890. Numbers to the right of the photographs indicate the percentage of cells with mitochondrial-localized Bax. C: control

Two additional Hsp90 inhibitors were tested: HSP990 and AUY. In combination with SCH, both inhibitors induced apoptosis of OS cells as effectively as did AAG and DMAG but at much lower concentrations (50 nℳ) (Figures 3a–c). As additional methods of monitoring apoptosis, we show caspase-3 activation (Figure 3d) and PARP (poly(ADP-ribose) polymerase) cleavage (Figure 3e) in OS1002 cells cotreated with SCH and AUY. Caspases become active when cleaved and disrupt cell function to elicit cell death; PARP is a caspase-3 substrate.^20^ Bax, a member of the Bcl-2 family of apoptosis regulatory proteins, perforates the outer mitochondrial membrane to release cytochrome c, which indirectly activates caspase-3.^21^ Bax is cytosolic in healthy cells and translocates to mitochondria in response to apoptotic signals. We show that Bax accumulates in mitochondria of OS1003 cells cotreated with SCH and AUY as evidenced by its colocalization with the mitochrondrial dye MitoTracker (Figure 3f). Colocalization was observed in 40.9% of cotreated cells as compared with 2.5%, 10.6% and 6.1% of control, SCH-treated and AUY-treated cells, respectively. Together, the data in Figures 2 and 3 show that SCH and Hsp90 inhibitors collaboratively induce robust apoptosis of OS cells.

Hsp90 inhibitors also boosted the apoptosis of OS cells cotreated with roscovitine (20 *μ*ℳ) or flavopiridol (200 nℳ) (Supplementary Figures 2a and b). These agents inactivate CDKs 1, 2 and 9, as does SCH; roscovitine also inactivates CDK7, and flavopiridol is a pan-CDK inhibitor (CDKs 1, 2, 4, 6, 7 and 9).^22, 23, 24, 25^ Hsp90 inhibitors did not induce apoptosis when combined with PD0332991, which selectively targets CDK4 and CDK6 (Supplementary Figure 2c).^26^ This finding may be explained by redundant actions of PD0332991 and Hsp90 inhibitors: PD0332991 inactivates CDK4 and CDK6, and Hsp90 inhibitors downregulate CDK4 and CDK6.^27, 28^

**Figure 4:**
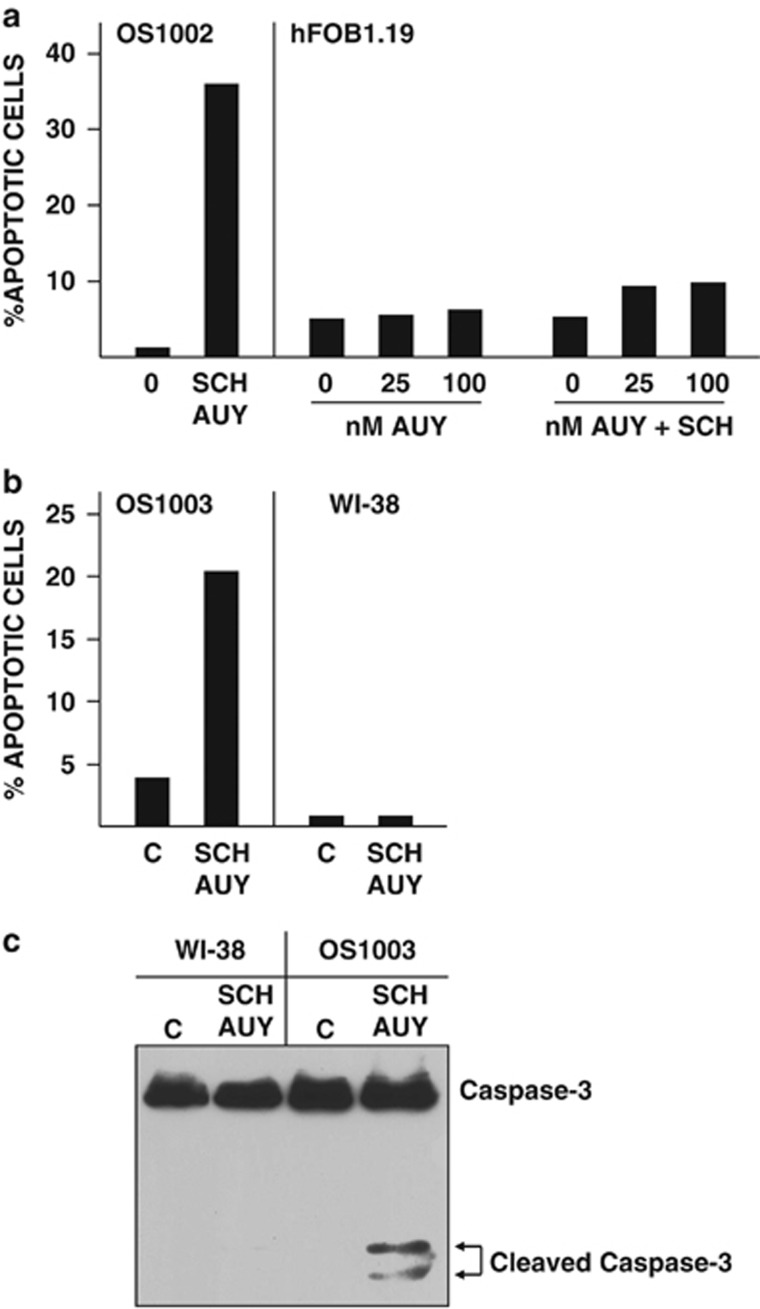
Survival of normal human osteoblasts and fibroblasts cotreated with SCH and AUY. (**a**) OS1002 and hFOB1.19 cells received 10 nℳ SCH and 25 or 100 nℳ AUY for 24 h. Percent apoptotic cells was determined by FACS analysis of annexin V-stained cells. (**b**, **c**) OS1003 and WI38 cells received 10 nℳ SCH and 100 nℳ AUY for 24 h. Percent apoptotic cells was determined by FACS analysis of annexin V-stained cells (**b**). Caspase-3 cleavage were determined by western blotting of cell extracts with antibody to caspase-3 (**c**). C: control

To be useful in chemotherapy, drugs must selectively kill tumor cells while sparing normal cells. Cotreatment of normal human osteoblasts (hFOB1.19) or fibroblasts (WI38) with SCH and AUY did not appreciably increase the percentage of annexin V-positive cells (Figures 4a and b) or induce caspase cleavage (Figure 4c). In contrast, 20 to 30% of cotreated OS cells were annexin V-positive (Figures 4a and b) and caspase cleavage was readily apparent (Figure 4c). Thus, the combination of SCH and AUY preferentially targets tumor cells rather than normal cells.

### SCH induces apoptosis of OS cells previously exposed to AUY

**Figure 5:**
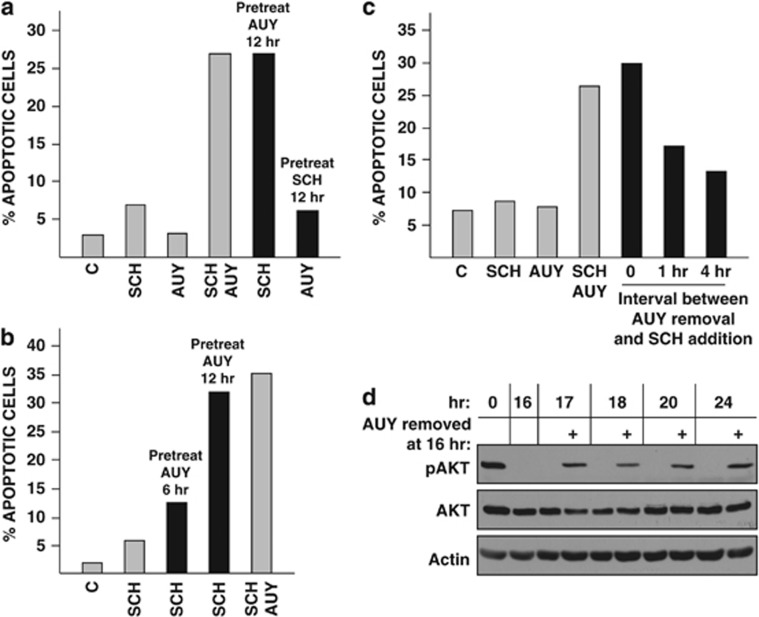
Sequential addition of AUY and SCH to OS cells. (**a**) OS1002 cells received 100 nℳ AUY or 10 nℳ SCH for 12 h (black bars). Untreated (gray bars) and pretreated cells were refed with fresh medium containing SCH, AUY or both and were harvested 36 h after refeeding. (**b**) OS1002 cells received 100 nℳ AUY for 6 or 12 h (black bars). Untreated (gray bars) and pretreated cells were refed with fresh medium containing SCH (10 nℳ) or SCH plus AUY and were harvested 36 h after refeeding. (**c**) Gray bars: OS1002 cells were refed with medium containing 10 nℳ SCH, 100 nℳ AUY or both and were harvested 48 h later. Black bars: OS1002 cells pretreated with AUY for 18 h were refed with fresh medium containing SCH or were incubated in medium alone for 1 or 4 h before addition of SCH. Cells were harvested 48 h after SCH addition. (**a–c**) Percent apoptotic cells was determined by FACS analysis of annexin V-stained cells. C: control. (**d**) OS1002 cells received 100 nℳ AUY for 16 h. Cells were refed with fresh medium with (+) or without AUY and were harvested at the indicated times. Amounts of AKT, phospho-AKT (pAKT) and actin (loading control) were determined by western blotting

We asked whether AUY and SCH induce apoptosis when added sequentially rather than simultaneously to cells. OS1002 cells received one inhibitor for 12 h, were refed with fresh medium containing the second inhibitor, and were harvested 36 h after refeeding. Cells did not apoptose when exposed to SCH and then to AUY (Figure 5a). They did, however, apoptose when the opposite was done: AUY followed by SCH was as effective as AUY and SCH together (∼27% apoptotic cells in both conditions).

AUY was maximally effective when applied to cells for 12 h but only marginally effective when applied to cells for 6 h (Figure 5b). Effectiveness also depended on the timing of SCH addition relative to AUY removal. In the experiment shown in Figure 5c, OS1002 cells received AUY for 18 h and were incubated in fresh medium for 0, 1 or 4 h before addition of SCH; cells were harvested 48 h after SCH addition. When SCH was added to cells immediately after AUY removal, 30% of the cells apoptosed. Delaying SCH addition for 1 h reduced the percentage of apoptotic cells to 17% delaying SCH addition for 4 h reduced it to 13%. Thus, sequential addition of AUY and SCH to OS cells is most effective when there is no gap between treatments.

Responsiveness of AUY-pretreated cells to SCH did not reflect the presence of residual AUY. AUY substantially reduced the phosphorylation (and thus the activity) of the Hsp90 client AKT; when AUY was withdrawn, phospho-AKT reaccumulated to near starting levels within 1 h (Figure 5d). Total amounts of AKT did not appreciably decline in cells receiving AUY for times up to 24 h; loss of AKT activity in the absence of changes in AKT abundance has also been observed in other systems.^29^

### SCH and AUY inhibit cell cycle progression

**Figure 6:**
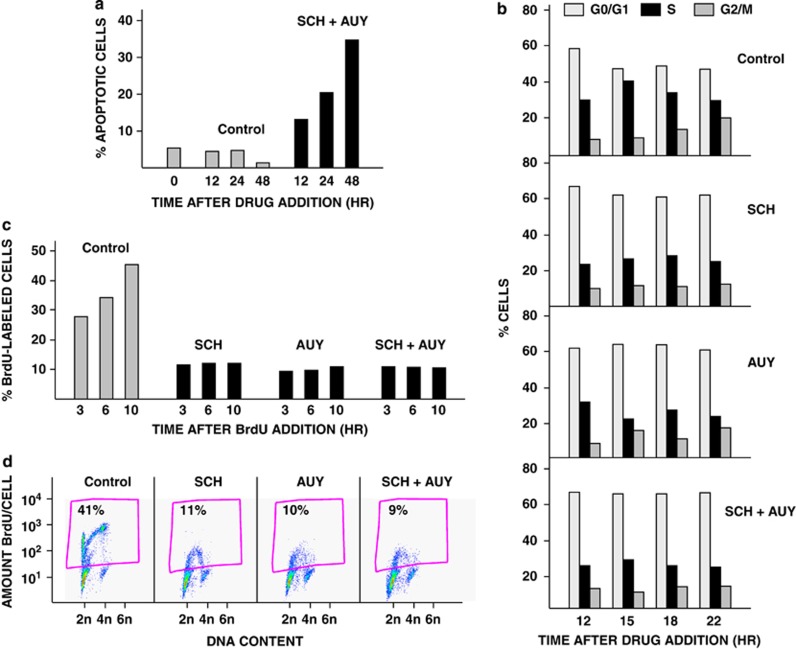
SCH and AUY inhibit cell cycle progression. (**a**) OS1002 cells received 20 nℳ SCH and 100 nℳ AUY for 12, 24 or 48 h. Percent apoptotic cells was determined by FACS analysis of annexin V-stained cells. (**b**) Cells received 20 nℳ SCH, 100 nℳ AUY, or both for 12, 15, 18 or 22 h. Cells were stained with propidium iodide, and cell cycle position was determined as described in Materials and Methods. (**c**) Cells received 20 nℳ SCH, 100 nℳ AUY, or both for 12 h. BrdU was added, and cells were harvested 3, 6 and 10 h after BrdU addition. Percent BrdU-labeled nuclei was determined as described in Materials and Methods. (**d**) Scatter plots for the 10 h time point in (**c**) are shown. The percentages of cells in S phase are indicated

OS cells begin apoptosing within 12 h of addition of SCH plus AUY (Figure 6a). We performed a cell cycle analysis to determine whether cells are cycling or arrested at this time. First, we quantified DNA content by propidium iodide staining. OS1002 cells received SCH, AUY or both for 12, 15, 18 or 22 h. Cell cycle distributions were the same for all three treatments and at all time points (Figure 6b). On average, percentages of drug-treated cells in G0/G1, S and G2/M were 65%, 25% and 13%, respectively. In comparison, percentages of control cells in G0/G1, S and G2/M were 52%, 35% and 15%, respectively. The small decrease in percentage of cells in S phase and accompanying increase in percentage of cells in G0/G1 in drug-treated as compared with control cultures suggest that SCH and AUY, alone and together, modestly inhibit S phase entry. We note that cells were attached to the plates at all time points.

Second, as part of the same experiment, we monitored DNA synthesis by bromodeoxyuridine (BrdU) incorporation. Cells received BrdU 12 h after drug addition and were harvested 3, 6 and 10 h later (these time points correspond to the 15, 18 and 22 h time points in Figure 6b). The percentage of control cells incorporating BrdU increased progressively from 28% at 3 h to 45% at 10 h (Figure 6c). In contrast, the BrdU-labeled component of SCH-treated, AUY-treated, and co-treated populations remained constant. Whereas 22–29% of drug-treated cells were in S phase as detected by propidium iodide (Figure 6b), only 9–12% of the drug-treated cells were BrdU-labeled (Figure 6c). These data suggest that cells do not actively synthesize DNA in the presence of SCH, AUY or both. Cotreatment of cells with SCH and AUY for 12 h did not induce histone H2AX phosphorylation (Supplementary Figure 3), a marker of double-stranded DNA breakage,^30^ and thus does not cause DNA damage.

Impaired S phase progression was not accompanied by a decrease in the percentage of G2/M cells (Figure 6b); this indicates that drug-treated cells do not exit G2/M. As further evidence, BrdU-positive cells accumulated in G0/G1 10 h after BrdU addition to control but not drug-treated cultures (Figure 6d). Collectively, our data suggest that SCH and AUY ‘freeze' cells in multiple phases of the cell cycle. We conclude that OS cells cotreated with SCH and AUY are not cycling at the onset of apoptosis. Cell cycle arrest, however, is insufficient or unrelated to apoptosis: cell cycle arrest occurs in response to either SCH or AUY, whereas efficient apoptosis requires both SCH and AUY.

### Combinatorial actions of SCH and AAG on other sarcoma cell cultures

**Figure 7:**
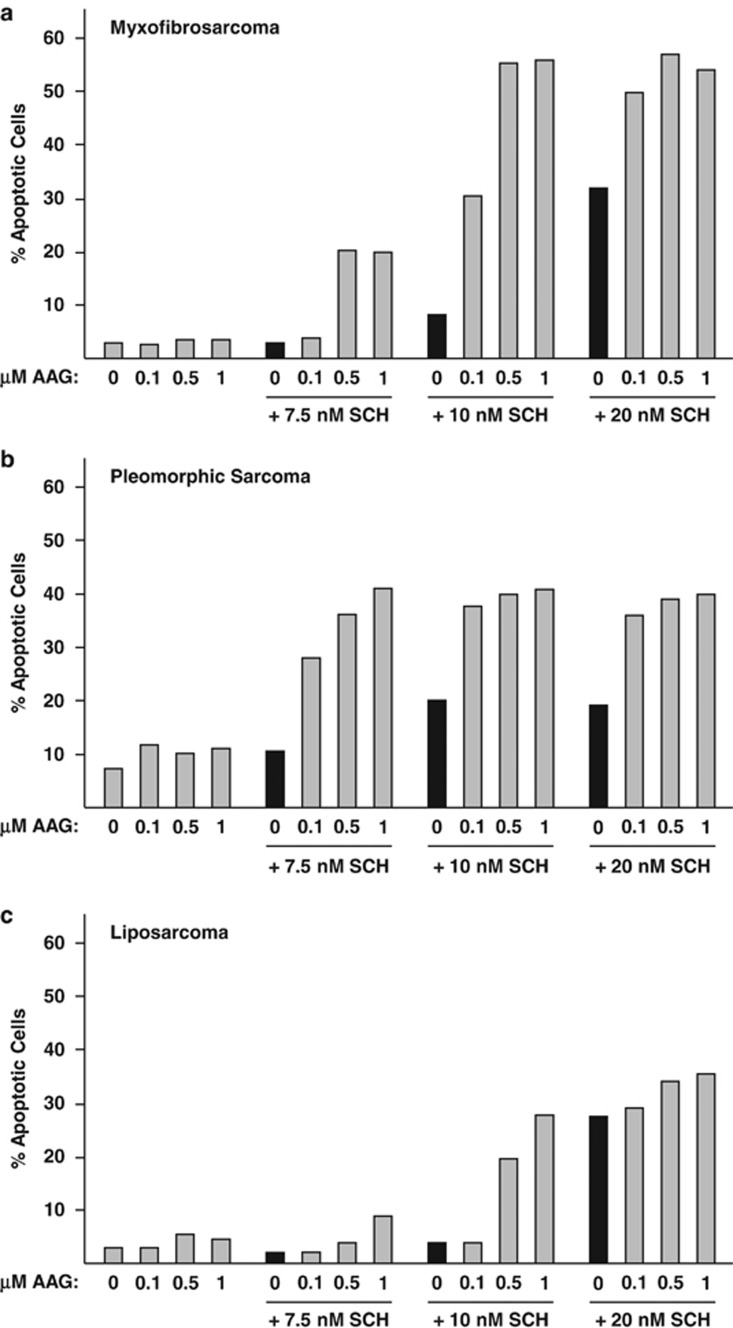
Effects of SCH and AAG on the survival of additional sarcoma cell cultures. (**a**–**c**). Cells received the indicated concentrations of SCH and AAG for 40 h. Percent apoptotic cells was determined by FACS analysis of annexin V-stained cells

We tested the effects of SCH and AAG on additional cultures prepared by us from resected sarcoma tissue: a low-grade myxofibrosarcoma (Figure 7a), a high-grade pleomorphic sarcoma with lung metastasis (Figure 7b) and a high-grade dedifferentiated liposarcoma (Figure 7c). All were refractory to AAG alone and more sensitive to SCH in the presence than in the absence of AAG. Myxofibrosarcoma cells were especially responsive to SCH plus AUY: more than 50% of cells apoptosed within a 40-hr treatment period. These findings extend the apoptotic actions of SCH and Hsp90 inhibitors to at least some other sarcomas in addition to OS.

## Discussion

Our studies show apoptosis of OS cultures by a novel drug combination: the CDK inhibitor SCH and an Hsp90 inhibitor. CDKs drive the cell cycle, which is often dysregulated in cancer, and ensure an ample supply of short-lived anti-apoptotic proteins. Hsp90 is essential for the proper functioning of numerous proteins and signal transduction pathways and is more susceptible to Hsp90 inhibitors in tumor cells than in normal cells. Thus, CDKs and Hsp90 are rational targets for drug intervention.

The OS cultures used in our experiments were derived from resected tumors and were passaged less than 20 times. Gillet *et al.*^31^ suggest that long-term culturing radically alters gene expression and distorts drug sensitivity: they found striking differences in the multi-drug resistance gene profiles of ovarian clinical samples *versus* established ovarian cancer cell lines. Thus, use of OS cultures minimizes potential culture-induced anomalies and increases the likelihood of obtaining information that will translate successfully to the clinic. We note that OS cultures are much less sensitive to SCH than are established OS cell lines such as U2OS and SaOs-2.^4^

We used four Hsp90 inhibitors in our experiments: AAG and DMAG, which are ansamycin derivatives, and HSP990 and AUY, which are synthetic small molecules classified as aminopyridine and resorcinol-containing, respectively.^32^ None induced OS apoptosis in the absence of SCH; all enhanced the weak apoptotic response elicited by SCH. HSP990 and AUY were especially potent, producing effects at concentrations of 25–50 nℳ. OS cells apoptosed when cotreated with SCH and an Hsp90 inhibitor regardless of whether their tumor of origin was localized or metastatic. Of particular note, OS1002 cells were derived from a tumor that recurred after chemotherapy. Combined application of SCH and Hsp90 inhibitor also induced the apoptosis of other sarcoma types but did not affect the survival of normal osteoblasts.

Interestingly, we show that AUY and SCH need not be present together to induce OS apoptosis: AUY can be added and removed before addition of SCH but not *vice versa*. This finding suggests that AUY elicits an event (or events) that renders OS cells responsive to SCH. For example, it may cause an anti-apoptotic protein to degrade or to lose its active conformation or may simply enervate cells by allowing build-up of toxic, unfolded protein aggregates.^33^ Sequential treatment was most effective when cells were primed with AUY for ∼12 h and immediately exposed to SCH. Thus, the AUY-induced priming event is complete by 12 h and is short-lived in the absence of AUY.

As a reverse scenario, it is possible that SCH renders OS cells responsive to AUY. AUY may elicit both apoptotic and anti-apoptotic signals; negation of the anti-apoptotic signal by SCH would allow apoptosis to proceed. A potential anti-apoptotic signal is the induction of Hsp72. Hsp72 is a member of the Hsp70 family of molecular chaperones, and Hsp90 inhibitors upregulate its expression by activating the Hsf-1 transcription factor.^34^ Colon cancer cells depleted of Hsp72 (and a second Hsp70 isoform), apoptosed when exposed to AAG, whereas mock-depleted cells did not.^35^ Similarly, Hsf-1-null transformed fibroblasts were more responsive to AAG than were their wild-type counterparts.^36^ Whether SCH prevents the upregulation of Hsp72 by Hsp90 inhibitors in OS cells remains to be determined. Regardless of the mechanism, sequential application of SCH and AUY may allow flexibility when planning treatment schedules. Previous studies showed apoptosis of Rb-positive breast cancer cells sequentially exposed to AAG and taxol; AAG was effective when added to cells less than 4 h before or after taxol.^37^

Our studies show that Bax is predominantly cytosolic in OS cells receiving SCH or AUY but accumulates in the mitochondria of cotreated cells. How SCH and AUY collaboratively trigger the mitochondrial translocation of Bax is unclear at present. Of interest is a recent study showing the interaction of Bax with the Hsp90 cochaperone p23 in the cytosol of healthy cells.^38^ Depletion or overexpression of p23 did not affect the subcellular location of Bax, thus suggesting that p23 controls functions of Bax unrelated to its location in the cell or that it modulates Bax location in concert with additional events.

In addition to inducing apoptosis, SCH and AUY also affected cell cycle progression. Alone or together, they blocked OS cells in G0/G1, S and G2/M. As robust apoptosis requires both inhibitors, cell cycle arrest is unrelated to or insufficient for the apoptosis of OS cells. Similar to our findings on OS cells, CDK inhibitors reduced the percentage of BrdU-positive S phase cells in myeloma, neuroblastoma, and colon, lung and breast carcinoma cultures; G2/M arrest, and in some instances G0/G1 arrest, was also observed.^39, 40, 41, 42, 43^ Hsp90 inhibitors arrest different tumor cell lines in different parts of the cell cycle: G0/G1 or G2/M, as shown by propidium iodide staining in several studies,^28, 44, 45, 46, 47, 48^ or G0/G1, G2 or M, as shown by Lyman *et al.*^49^ by combined use of a thymidine analog and cell cycle markers. As an exception, HCT116 colon carcinoma cells did not accumulate in either G0/G1 or G1/M in response to AAG.^46^ Thus, the cell cycle responses of tumor cells to Hsp90 inhibitors are variable and may reflect cellular genotype, at least in part.

Advances in surgical techniques and the advent of multi-agent chemotherapy have greatly improved the prognosis of OS patients. However, better treatments are still needed, particularly for patients with recurrent or metastatic disease. We suggest that SCH and AUY may be a promising strategy for treatment of OS and other types of sarcomas.

## Materials and Methods

### Cell culture

Fresh, finely minced sarcoma tissue was incubated with DNase I and collagenase D for 30 min at 37 °C, washed extensively and transferred to 10 cm^2^ culture flasks. Cells were incubated in a 1 : 1 mixture of Ham's F12 medium and Dulbecco's Modified Eagle's medium containing 10% fetal calf serum. After expansion of cultures, frozen stocks were prepared. Frozen stocks served as the source of the OS cultures used in experiments. OS cells were passaged less than 20 times. NHOst cultures were purchased from Lonza (Walkersville, MD, USA) and cultured in Osteoblast Growth Medium (OBM Bullet Kit, Lonza). hFOB1.19 cells (normal human fetal osteoblasts expressing temperature-sensitive SV40 large T antigen) were obtained from ATCC and cultured at the permissive temperature (33.5 °C) in a 1 : 1 mixture of Ham's F12 medium and Dulbecco's Modified Eagle's medium containing 0.3 mg/ml G418 and 10% fetal calf serum but lacking phenol red. WI38 cells (normal human fibroblasts) were from our frozen stocks and were cultured in minimum essential medium containing 10% fetal calf serum.

### RT-PCR

Total cellular RNA was isolated using TRIzol reagent and used as a template for cDNA synthesis using the SuperScript First Strand Synthesis System (Invitrogen, San Diego CA USA). Primer sequences for alkaline phosphatase were: 5′—ACGTGGCTAAGAATGTCATC—3′ and 5′—CTGGTAGGCGATGTCCTTA—3′. Primer sequences for osteopontin were: 5′—CCAAGTAAGTCCAACGAAAG—3′ and 5′—GGTGATGTCCTCGTCTGTA—3′. Primer sequences for osteocalcin were: 5′—ATGAGAGCCCTCACACTCCTC—3′ and 5′–GCCGTAGAAGCGCCGATAGGC—3′. Primer sequences for GAPDH were: 5′—ACCCAGAAGACTGTGGATGG—3′and 5′—TCTAGACGGCAGGTCAGGTC—3′. PCR products were run on 2% agarose gels and visualized by ethidium bromide staining.

### Annexin V binding

Cells were detached from plates with trypsin-EDTA and combined with floating cells. Cells were stained with Annexin V-FITC and PI/7-AAD (BD Pharmingen, San Diego, CA, USA) and analyzed by FACS as described previously.^50^ Results shown in bar graphs were taken from the lower right quadrant of the plots and represent early apoptosis.

### Western blotting

Cells were washed with ice-cold phosphate-buffered saline (PBS), scraped into PBS and collected by centrifugation. Pellets were resuspended in a lysis buffer containing 50 mℳ Hepes, 150 mℳ NaCl, 1 mℳ EDTA, 1 mℳ EGTA, 10% glycerol, 0.5% Nonidet P-40, 0.5% Tween-20, 1 mℳ dithiothreitol, and protease inhibitor cocktail (Sigma, St Louis, MO, USA) and vortexed for 20 min at 4 °C; insoluble material was removed by centrifugation. Proteins were resolved by SDS-polyacrylamide gel electrophoresis and transferred to nitrocellulose membranes. Membranes were incubated sequentially in Tris-buffered saline containing 0.05% Tween-20 and 5% non-fat dry milk as follows: no addition, 1 h at room temperature (blocking); primary antibody, overnight at 4 °C; and secondary antibody, 1 h at room temperature. Bound secondary antibody was detected using WestPico and WestFemto chemiluminescent substrates (Pierce, Rockford, IL, USA).

### Immunostaining

Cells on glass chamber slides received 40 nℳ MitoTracker Red (Invitrogen) 30 min before harvest. Cells were washed with PBS, fixed for 20 min in PBS containing 4% paraformaldehyde and permeabilized for 5 min in PBS containing 0.1% Triton X-100. Fixed, permeabilized cells were blocked with 1% bovine serum albumin and incubated with anti-Bax mouse monoclonal antibody (6A7, Santa Cruz, CA, USA) for 2 h at 37 °C. Cells were washed with PBS and incubated with goat anti-mouse IgG conjugated to Alexa Fluor-488 for 30 min at 37 °C. Stained slides were mounted with Vectashield mounting medium containing DAPI (Vector Laboratories, Burlingame, CA, USA). Fluorescent images were visualized by confocal microscopy.

### Propidium iodide staining

Cells were removed from the plates with 0.125% trypsin and 0.5 mℳ EDTA in PBS; an equal volume of medium containing 10% serum was added to neutralize the trypsin. Cells were pelleted and resuspended in PBS (1 ml), and 100% ethanol (3.5 ml) was added slowly. Cells were incubated at 4 °C for a minimum of 16 h, pelleted, and resuspended in PBS containing 0.1% Tween-20, 0.05% bovine serum albumin, 10 mg/ml RNase A and 50 mg/ml propidium iodide. After a further incubation at 4 °C for at least 4 h, cell cycle distribution was determined by FACS. DNA content (FL3: FL4 area) and relative amount of BrdU per cell (FL1:H Anti-BrdU-FITC) are plotted on the x and y axes, respectively, of the scatter plots.

### BrdU staining

Cells received 30 *μ*ℳ BrdU for times indicated in the figure legends. Cells were removed from the plates and fixed in PBS and ethanol as described above for propidium iodide staining. Pelleted cells were resuspended in 2 𝒩 HCl and incubated for 30 min at 37 °C to denature DNA. Sodium borate (final concentration, 0.1 ℳ) was added to neutralize the pH, and cells were washed thoroughly to remove any residual acid. Cells were incubated overnight at 4 °C in PBS containing 0.1% bovine serum albumin, 0.1% Tween-20 and a 1/20 dilution of mouse monoclonal anti-BrdU-FITC antibody (eBioscience, San Diego, CA, USA). After washing, cells were incubated in PBS containing 0.1% Tween-20, 0.05% bovine serum albumin, 10 mg/ml RNase A and 50 mg/ml propidium iodide staining for at least 4 h at 4 °C. Percent BrdU-labeled cells and DNA content were determined by FACS.

### Reagents

Antibodies to PARP, caspase-3, AKT, pAKT and pERK were obtained from Cell Signaling Technology (Beverly, MA, USA). Antibody to actin was obtained from Sigma. AAG and DMAG were purchased from LC Laboratories (Woburn, MA, USA) and InvivoGen (San Diego, CA, USA), respectively. AUY and HSP99 were provided by Novartis (East Hanover, NJ, USA), and SCH was provided by Merck/Schering-Plough Corporation (North Wales, PA, USA) and CTEP.

### Statistical analysis

*P*-values were determined by a paired, two-tailed Student's *t*-test of three or more samples. Treated groups were compared with untreated (control) groups. *P*-values<0.05 are considered significant.

## Abbreviations

AAG: 17-allylamino-17-demethoxygeldanamycin
AUY: NVP-AUY922
BrdU: bromodeoxyuridine
CDK: cyclin-dependent kinase
DMAG: 17-(2-dimethylaminoethyl) amino-17-demethoxygeldanamycin
FACS: fluorescence-activated cell sorting
Hsp: heat shock protein
MAP: methotrexate, cisplatin, and doxorubicin
HSP990: NVP-HSP990
PARP: poly(ADP-ribose) polymerase
PBS: phosphate-buffered saline
OS: osteosarcoma
SCH: SCH727965
