# Supplementary material for: Apoptosis of osteosarcoma cultures by the combination of the cyclin-dependent kinase inhibitor SCH727965 and a heat shock protein 90 inhibitor
Source: Cell Death Dis. 2013 Mar 28;4(3):e566–. doi: 10.1038/cddis.2013.101 (PMC3613821; doi:10.1038/cddis.2013.101)
Supplement: Supplementary Data [file cddis2013101x1.pdf]

Supplemental Data

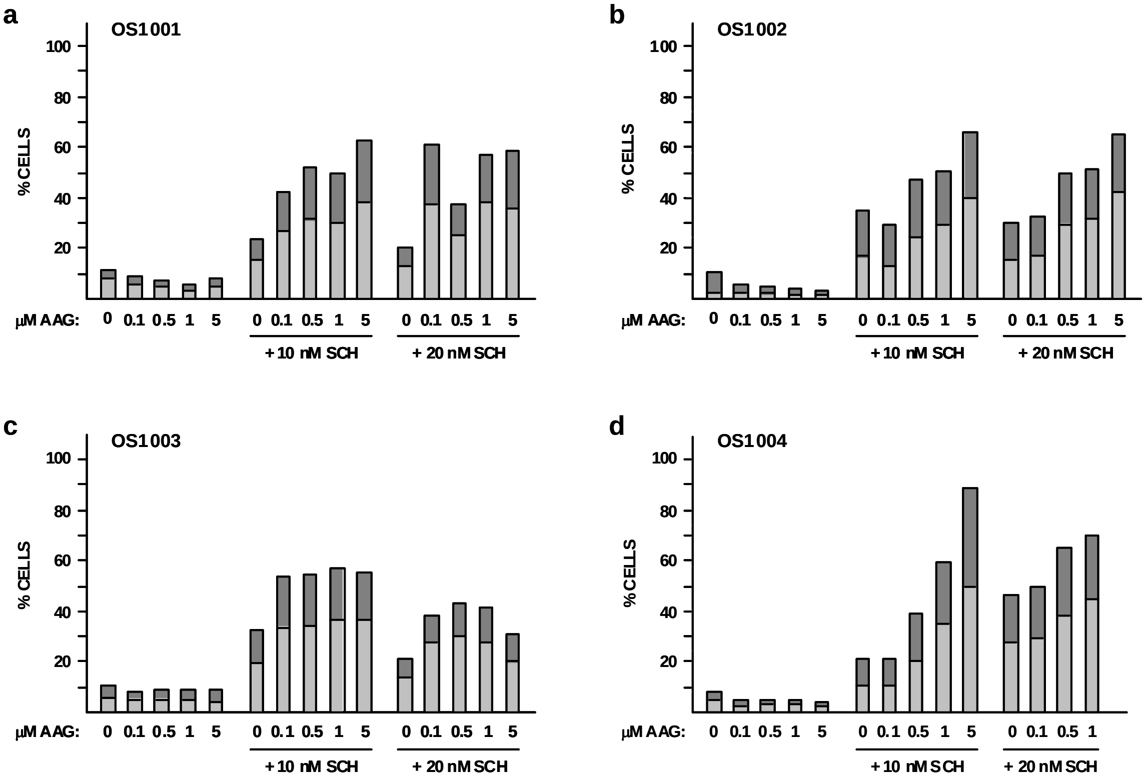

Supplemental Figure 1. Early apoptosis and late apoptosis/necrosis of OS cells co-treated with SCH and AAG. (**a-d**) Cells received the indicated concentrations of SCH and AAG for 48 hr. Cells were stained with Annexin V-FITC and PI/7-AAD and analyzed by FACS. Light gray bars: percent cells in early apoptosis (lower right quadrant of the plots). Dark gray bars: percent cells in late apoptosis or necrosis (upper right quadrant of plots).

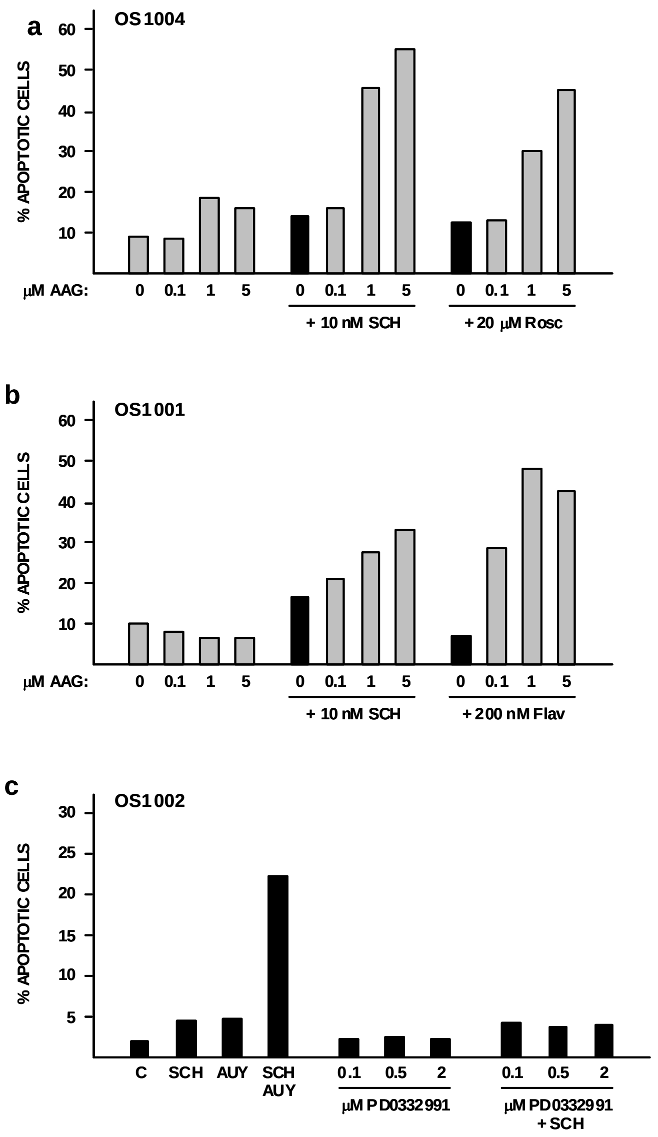

Supplemental Figure 2. Effects of CDK inhibitors and Hsp90 inhibitors on OS survival. (**a**) OS1004 cells received various concentrations of AAG and either 10 nM SCH or 20 µM roscovitine (Rosc) for 48 hr. (**b**) OS001 cells received various concentrations of AAG and either 10 nM SCH or 200 nM flavopiridol (Flav) for 48 hr. (**c**) OS1001 cells received 10 nM SCH, 100 nM AUY, and various concentrations of PD0332991 as indicated for 48 hr. (**a-c**) The percentage of cells in early apoptosis was determined by FACS analysis of annexin V-stained cells.

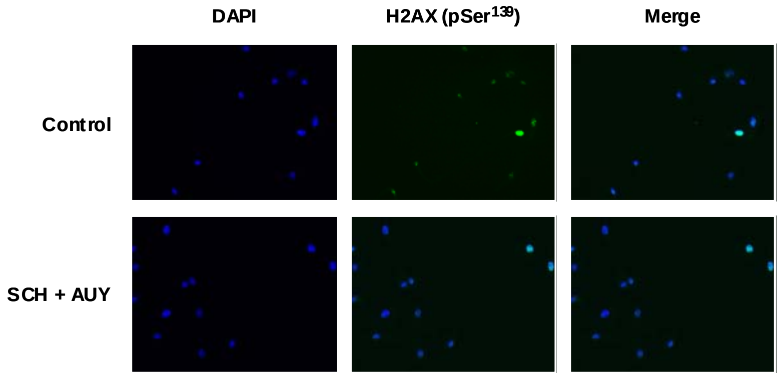

Supplemental Figure 3. Effects of inhibitors on H2AX phosphorylation. OS1002 cells on glass coverslips in 6-well plates received 20 nM SCH and 100 nM AUY for 12 hr. Cells were fixed with 4% paraformaldehyde for 15 min at room temperature and permeabilized with 0.2% Triton X-100 in phosphate-buffered saline for 10 min at room temperature. After a 1 hr incubation in blocking buffer (phosphate-buffered saline containing 0.1% Triton X-100, 3% bovine serum albumin, and 10% goat serum), cells were immunostained overnight at 4°C with a 1/400 dilution of anti-phospho-histone H2AX (serine 139) antibody (Cell Signaling) in blocking buffer. Primary antibody staining was developed with secondary antibody conjugated to Alexa Fluor-488, and coverslips were mounted with Vectashield mounting medium containing DAPI (Vector Laboratories). Cells were analyzed with a Zeiss upright fluorescent microscope at 200× magnification. A representative field is shown. Percentages of control and treated H2AX-positive cells are 12.6 and 10.4, respectively.
